# Bacteriophage and Bacterial Susceptibility, Resistance, and Tolerance to Antibiotics

**DOI:** 10.3390/pharmaceutics14071425

**Published:** 2022-07-07

**Authors:** Qingquan Chen, Tejas Dharmaraj, Pamela C. Cai, Elizabeth B. Burgener, Naomi L. Haddock, Andy J. Spakowitz, Paul L. Bollyky

**Affiliations:** 1 Division of Infectious Diseases and Geographic Medicine, Department of Medicine, Stanford University School of Medicine, Beckman Center, 279 Campus Drive, Stanford, CA 94305, USA; tdharma1@stanford.edu (T.D.); nhaddock@stanford.edu (N.L.H.); pbollyky@stanford.edu (P.L.B.); 2 Department of Chemical Engineering, Stanford University, Stanford, CA 94305, USA; pamcai@stanford.edu; 3 Center for Excellence in Pulmonary Biology, Department of Pediatrics, Stanford University, Stanford, CA 94305, USA; eburgener@stanford.edu (E.B.B.); ajspakow@stanford.edu (A.J.S.)

**Keywords:** bacteriophage, antibiotics resistance, horizontal gene transfer, mobile gene elements, phage antibiotics synergy

## Abstract

Bacteriophages, viruses that infect and replicate within bacteria, impact bacterial responses to antibiotics in complex ways. Recent studies using lytic bacteriophages to treat bacterial infections (phage therapy) demonstrate that phages can promote susceptibility to chemical antibiotics and that phage/antibiotic synergy is possible. However, both lytic and lysogenic bacteriophages can contribute to antimicrobial resistance. In particular, some phages mediate the horizontal transfer of antibiotic resistance genes between bacteria via transduction and other mechanisms. In addition, chronic infection filamentous phages can promote antimicrobial tolerance, the ability of bacteria to persist in the face of antibiotics. In particular, filamentous phages serve as structural elements in bacterial biofilms and prevent the penetration of antibiotics. Over time, these contributions to antibiotic tolerance favor the selection of resistance clones. Here, we review recent insights into bacteriophage contributions to antibiotic susceptibility, resistance, and tolerance. We discuss the mechanisms involved in these effects and address their impact on bacterial fitness.

## 1. Introduction

The modern era of antibiotics started with the discovery of penicillin by Sir Alexander Fleming in 1928 [1]. In the following decades, advances in drug screening, chemical synthesis, and manufacturing led to the wide availability of several classes of highly effective antimicrobial agents and a well-developed commercial pharmaceutical industry. Antimicrobial therapy rapidly became a cornerstone of human healthcare.

Bacterial geneticists initially believed that the development of widespread antimicrobial resistance (AMR) was unlikely. However, this view failed to appreciate the facility with which bacteria exchange genetic information [2,3,4], including the horizontal transfer of AMR [5,6]. Researchers also failed to consider the role of antimicrobial tolerance—the ability of metabolically dormant bacteria and bacteria sheltered within biofilms to evade antibiotics—as a gateway for the development of AMR.

Unfortunately, widespread AMR to many classes of antibiotics is now prevalent and is a major threat to human health [7]. In the United States alone, more than 2.8 million infections and 35,000 deaths per year are attributable to AMR bacteria [8]. Globally, at least 1.2 million people died in 2019 because of bacterial AMR infections [9]. The total number of deaths attributable to AMR organisms is expected to reach 10 million globally by 2050 [9].

Despite the advancements in biotechnology, genetic engineering, and synthetic chemistry, antibiotic development has failed to keep pace with the spread of AMR [10]. There is, therefore, great interest in identifying factors that impact antibiotic treatment failures as well as therapies that can complement or substitute for antibiotics [11].

Given this need, there is a resurgence of interest in bacteriophages (phages), viruses that parasitize bacteria [12,13]. Before the discovery of penicillin, phages were discovered independently in 1915 by Frederick Twort, a British pathologist [14], and in 1917 by Félix d’Hérelle, a French-Canadian microbiologist [15]. Despite the great promise of phages as antibacterial agents, penicillin and other antibiotics were more successful. For many decades, research into the clinical applications of phages was largely abandoned in North America and Western Europe [16,17]. The spread of AMR and lack of antibiotic development has led to renewed interest in phages.

Phages employ one of several reproductive strategies. Lytic phages are obligate pathogens of bacteria that lyse their bacterial hosts upon replication [18]. Lysogenic phages can integrate within the bacterial genome and lyse their hosts opportunistically [19]. A sub-set of lysogenic phages (notably inoviruses) emerge from their bacterial hosts without lysis; this is called chronic infection [20,21]. Each of these phage reproductive strategies has distinct impacts on bacterial biology [22] in ways that may influence antimicrobial therapy.

In addition, over the past 15 years, the interest in using bacteriophage therapy has been re-kindled in laboratories and hospitals. Despite mixed results from phage therapy clinical trials [23,24,25], there are multiple instances and case series of successful phage therapy using either conventional or modified phages [26,27,28,29,30]. Phage therapy is generally safe and well-tolerated [31], although many questions remain regarding the optimal dosages and treatment regimens [32].

Bacteriophages impact bacteria in ways that intersect with how conventional antibiotics impact bacteria. Like penicillin and many other early antibiotics, phages have a long evolutionary history with bacteria. Phages exert strong selective pressures on bacteria and have major roles in the transfer of genetic material between bacterial strains and species [33,34,35,36]. This review focuses on the contributions of phages to bacterial tolerance and resistance development to conventional pharmaceutical antibiotics. In particular, we review recent insights into lytic phage contributions to antibiotic susceptibility, lytic and lysogenic phages to overcome antibiotic resistance, and filamentous phages to treat antibiotic tolerance. We discuss the mechanisms involved in these effects and address their impact on bacterial fitness and antimicrobial therapy.

## 2. Lytic and Lysogenic Phages Contribute to AMR

AMR occurs when inherited mutations in bacteria cause the drugs used to treat infections to become less effective [9,37]. The effects of such mutations are measured by minimum inhibitory concentration (MIC), which is the lowest concentration of antibiotics required to inhibit bacteria growth [38]. As a group, bacteria are not uniformly susceptible or resistant to different antibiotics. The level of susceptibility depends on the composition of bacteria, which leads to a range of MIC for bacterial species. The average MIC is viewed as a convenient metric to evaluate the resistance of tested bacteria.

**Figure 1:**
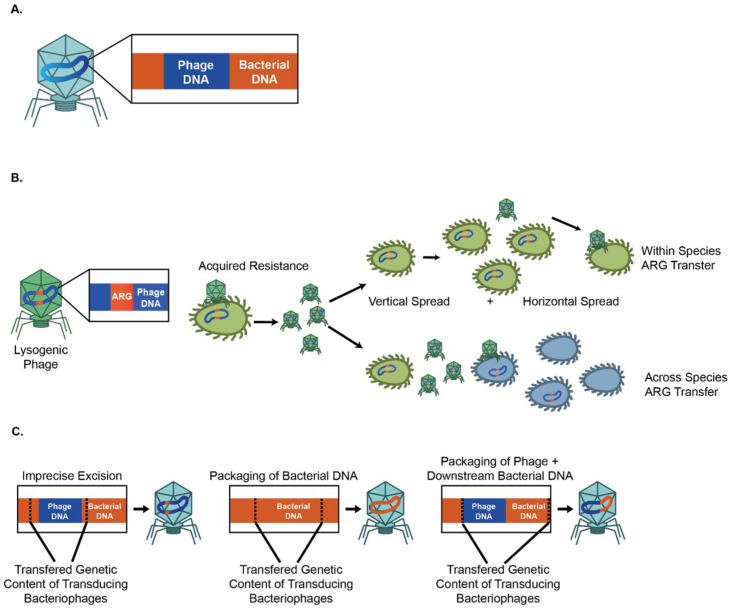
Lytic and lysogenic phages can contribute to bacterial antimicrobial resistance. (**A**) Bacteriophages can carry MGEs and mediate ARG movements. (**B**) Lysogenic bacteriophages contribute to the vertical and horizontal spread of ARGs. (**C**) Three main mechanisms for the phage-mediated spread of genetic material.

AMR is heritable and is mediated by the presence of antibiotic resistance genes (ARGs). Bacteria acquire ARGs in multiple ways [39]. One of the ways is that bacteria acquire antibiotic resistance via horizontal transfer of ARGs between individual bacteria or between bacterial species, which can be mediated by bacteriophages, via vertical transfer of ARGs to daughter bacteria, or through de novo chromosomal mutations. There is, therefore, a need to understand the mechanisms, frequency, reservoirs, and vectors governing the horizontal transfer of AMR (Figure 1A).

Although most AMR does not originate or spread via phages, there are indications that bacteriophages can be involved in the transfer of AMR. Here we review that literature.

### 2.1. Horizontal Transfer of Mobile Genetic Elements (MGEs) Promotes the Acquisition and Spread of ARG

Horizontal gene transfer, the movement of genetic material between organisms, is responsible for the dissemination of ARGs [40]. It allows bacteria to acquire new genetic material from outside their clonal lineage. Because of its ability to transfer genetic elements, horizontal gene transfer contributes significantly to the spread of bacterial AMR. Horizontal gene transfer has been extensively covered in several excellent reviews [40,41,42,43]. Horizontal gene transfer is mediated by mobile genetic elements (MGEs), such as bacteriophages and plasmids, which provide an important resource for bacterial genetic diversity as well as bacterial evolution [43,44] (Figure 1B). MGEs mediate the movement of genetic material within genomes or between bacterial hosts [43]. Several comprehensive reviews of MGEs are available [40,43]. The acquisition of ARGs is facilitated by the horizontal gene transfer [45,46] of MGEs, including plasmids [47], transposons, and integrons, through conjugation [48] and viral transduction [48,49]. In a later section, we will further discuss how phages contribute to the acquisition of ARGs. Most evolutionary models consider MGE-mediated horizontal transfer of ARGs from a cost–benefit perspective. Plasmids and other MGEs are an efficient means of exchanging genetic information. However, these elements are still costly as they necessitate the synthesis of proteins (such as conjugation pili), RNA, and DNA, which incur a fitness cost [50,51]. Further, MGEs often integrate into chromosomes, thereby potentially disrupting important genes [52]. However, this cost can be surmounted by other adaptive or addictive traits, such as antibiotic resistance [40,53,54].

### 2.2. Lysogenic Bacteriophages Can Contribute to the Vertical and Horizontal Spread of ARGs

Lysogenic (temperate) phages can integrate into the bacterial genome as prophages or persist as an extrachromosomal plasmid [55]. However, prophages can also be induced to undergo lytic replication at times of bacterial stress.

Many prophages carry ARGs [56,57,58,59,60,61]. A longitudinal study of viromes from human fecal samples found that antibiotic resistance genes were highly abundant among phage genomes [62]. In another study, 77% of 80 fecal samples from healthy individuals showed that they harbor at least one ARG [63]. Resistance genes including *bla*_TEM_, *bla*_CTX-M-1_, *mecA*, *armA*, *qnrA*, and *qnrS* were identified; *bla*_TEM_, *qnrA*, and *bla*_CTX-M-1_ were the most abundant, and *armA*, *qnrS*, and *mecA* were less prevalent [63]. High levels of ARGs were likewise reported in phages from the airways of individuals with cystic fibrosis (CF) [64,65] and feces samples of antibiotic-treated mice [66]. Moreover, in an ex vivo study, phages isolated from antibiotic-treated mice were transferred to aerobically cultured naïve microbiota and found to increase the frequency of drug resistance isolates in naïve microbiota compared to cultures infected with phages from untreated mice [66].

Phages also transfer ARGs to the environment. Lekunberri et al. analyzed 33 viromes sampled from diverse habitats, including human and pig feces, raw sewage, fresh water, and marine environments from public repositories, finding that human-associated viromes do not contain ARGs, while six pig-associated viromes harbored a high abundance of ARGs [59]. Phages from sewage and aquatic environments from around the world have a high diversity of ARGs [59], and studies have shown that aquatic phages serve as reservoirs for ARGs [67,68].

The presence of ARGs in lysogenic phages is consistent with strategies employed by phages to ensure their maintenance within the bacterial genome [55,69,70,71,72,73]. Prophages often express genes that provide competitive advantages to their host, including genes involved in bacterial pathogenicity [74,75,76] or virulence factors [77,78,79,80,81,82]. Prophages also have various mechanisms to prevent infection by other phages [83,84,85,86]. However, the role of phages in the spread of ARGs remains controversial. Enault et al. pointed out that estimates of phage-mediated ARG transfer could be too high due to excessive bacterial DNA content as well as inflated false positives because of the relaxed threshold in in silico detection of ARGs [87]. Instead, Enault et al. suggested that to carefully quantify the bacterial DNA contamination, use a conservative threshold to quantify bona fide ARGs, and apply a discovery-based work process with a manual inspection to remove false positive hits in ARGs. These steps may help properly estimate the role of phages in the spread of ARGs.

The relative importance of phages as a mechanism of horizontal transfer of ARGs is also unclear. The cost/benefit relationship of phages to bacteria is more complex than for plasmids or other MGEs, as the benefits conferred by genes associated with prophages are offset by the threat of bacterial lysis. However, lysis might also help transfer ARGs to neighboring bacterial populations during times of stress, such as during antibiotic treatment. In this way, the lytic portion of the lysogenic phage life cycle might benefit the rest of the bacterial population in some settings [88].

### 2.3. Both Lytic and Lysogenic Phages Can Promote Dissemination of ARGs via Transduction

Phages can also spread ARGs via transduction, a process in which bacterial DNA is packaged into phage particles during lysis with progeny phages to infect new susceptible bacterial hosts. [89,90]. Phages thereby help ensure the efficient transfer of DNA to appropriate hosts [91,92,93,94]. ARGs can be mobilized by both lytic and lysogenic phages [95].

Three methods of phage-mediated transduction have been identified (Figure 1C). First, specialized transduction is mediated by temperate phages, which inadvertently mobilize host genes adjacent to phage insertion sites as a result of imprecise excision [96]. Second, generalized transduction occurs when bacterial DNA, instead of phage DNA, is packaged into the phage head [96]. Given this ability to package large fragments of DNA, transduction can indirectly mediate the transfer of ARGs associated with other MGE. Zhang et al. showed that T4-like phage misloaded plasmid-borne ARGs by generalized transduction [97]. Transduction can mediate the transfer of ARGs between bacterial species as well. Studies have likewise shown that polyvalent phages disseminate ARGs between several *Enterococcus* [98] and *Staphylococcus* [99] species under laboratory conditions. Evidence suggests that phages contribute to the recombination of ARGs such as *bla*_CTX-M_, *mel*, and *tetM* across multiple bacteria genera, including *S. enterica*, *E. coli*, *S. pneumoniae*, and *S. sonnei* [100,101]. Although phages can mediate horizontal bacterial DNA exchange via specialized and generalized transduction, these processes are relatively inefficient. The frequencies of these processes are low, and the transfer of ARGs is dependent on antibiotic resistance genes immediately flanking phage insertion sites and imprecise excision in specialized transduction [88].

The third and most recently discovered form of phage-mediated transduction is lateral transduction. Here, newly generated phage capsids package predominantly bacterial DNA downstream of the phage insertion site with high efficiency [102]. Lateral transduction is the most powerful mode of phage-mediated DNA transfer, capable of transferring several hundred kilobases and a large span of the bacterial genome [102]. Instead of using the *ppac* sites as in generalized transduction, lateral transduction uses embedded *pac* sites for DNA packaging. Recently, Humphrey et al. used *S. aureus* and *Salmonella* spp. as reference organisms and showed that chromosomally encoded bacterial genes could be transferred at up to 1000-fold higher rates by lateral transduction than generalized transduction [103].

Conjugation involving plasmids is perhaps the best-understood route of horizontal gene transfer [104,105]. Studies showed that phages could potentially inhibit bacterial conjugation and potentially reduce ARG dissemination [106]. However, a recent study showed that when phages infect SXT -containing *V. cholerae*, high-frequency conjugative transfer of SXT ICEs is induced, leading to the dissemination of both phage and antibiotic resistances. Similarly, coliphage could also stimulate higher frequency conjugation of ICEs from an *E. coli* donor to a *V. cholerae* recipient [107].

## 3. Bacteriophage and Antimicrobial Tolerance

Antimicrobial tolerance was first coined by Horne et al. [108]. Later, Kester and Fortune defined antimicrobial tolerance as a population-level phenomenon that enables the population to survive the duration of a transient antibiotic treatment several times above the MIC without a resistance mechanism [38,109]. Unlike AMR, antimicrobial tolerance has a distinct mechanism to escape antibiotic-mediated killing [110]. In addition to occasional mutations, antimicrobial tolerance can result from metabolic adaptations [111] or biofilm production [112] and does not confer a higher MIC to the descendants of bacterial survivors [113]. Moreover, antimicrobial tolerance has been shown to increase antimicrobial resistance [114,115,116]. Hence, it is important to understand how bacteriophages contribute to antimicrobial tolerance to prevent further resistance development.

### Filamentous Bacteriophages Contribute to Bacterial Tolerance by Promoting Biofilm Production

A bacterial biofilm is a complex structure that adheres to biological or non-biological surfaces [117]. Biofilms encapsulate bacteria with a matrix that includes polysaccharides (e.g., alginate) and eDNA, as well as bacterial proteins [118].

Bacterial biofilms promote antibiotic tolerance, the ability of bacteria to proliferate despite treatment with antimicrobial agents, by preventing antibiotics from penetrating and then reaching target bacteria [119,120,121,122,123,124,125]. Over time, this tolerance is thought to select for antimicrobial resistance [126,127,128]. Similar effects may also characterize sputum colonized by bacteria. There are reports that the sputum of individuals with CF binds positively charged antibiotics and reduces their efficacy against *P. aeruginosa* [129,130].

Pf phage harbored by *P. aeruginosa* contributes to *P. aeruginosa* biofilm formation by Pf positive strain [131]. Filamentous phages belong to a subgroup of the family *Inoviridae*, and are long, thin phages ranging from 800 nm to 4 µm in length [20,132]. They are broadly distributed and can infect both Gram-positive and Gram-negative bacteria [133]. Most filamentous phages are lysogenic but extrude progeny phages from the bacterial cell without lysis in a cycle known as chronic infection [20,21]. Hence, when isolated, filamentous phages do not form clear plaques like lytic phages, but instead, form opaque zones of reduced growth that resemble the turbid plaques of lysogenic phages [55]. As with other lysogenic phages, filamentous phages influence the virulence of hosts by transferring genetic material through horizontal and vertical transmission [134,135].

**Figure 2:**
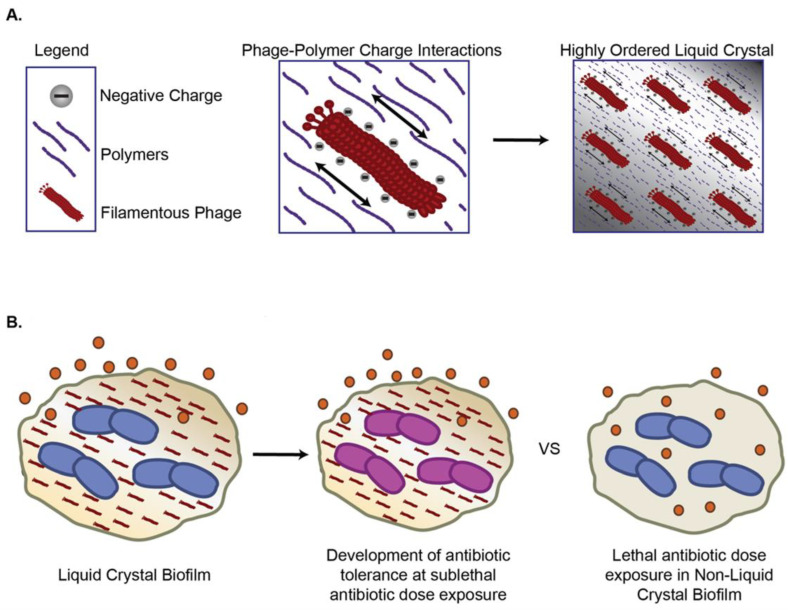
Filamentous bacteriophages (*Inoviruses*) increase antimicrobial tolerance via (**A**) Charged filamentous phages organize polymers into liquid crystal biofilms; (**B**) Liquid crystal biofilms sequester antibiotics and promote development of antibiotic tolerance at sublethal doses.

Pf phages are widespread among *P. aeruginosa* [136]. During biofilm growth, Pf genes are among the most upregulated, with a 100–1000 fold increase in expression relative to the planktonic growth mode [137]. Proteomic studies identified that Pf genes were a major portion of the most upregulated genes during anaerobic growth conditions that mimic those of the lungs of individuals with CF [138]. Rice et al. reported that Pf phages contribute to the *P. aeruginosa* biofilm formation and virulence. Building on this work, Secor et al. reported that Pf4 promotes the organization of human and microbial biofilm polymers into a liquid crystal [72,139,140,141] (Figure 2A). These effects are mediated by charge-based interactions between phages and polymers, contributing to the adhesivity and viscosity of *P. aeruginosa* biofilms [142] and promoting bacterial aggregation [143]. Fd phage, a filamentous phage from *E. coli*, promotes similar structures [142], while a related filamentous phage produced by Neisseria meningitides promotes bacterial colonization to apical surfaces of host epithelial cells [144].

Pf phage and liquid crystalline biofilms hinder antibiotic penetration, thereby promoting antibiotic tolerance [72,142] (Figure 2B). In particular, Pf phage increase *P. aeruginosa* tolerance to tobramycin, gentamicin, and colistin [145]. Tarafder et al. demonstrated that liquid crystalline phage droplets also form occlusive compartments around bacteria that shield them from antibiotics [145]. The potential exclusion could be mediated by either limiting antibiotics diffusion or excluding antibiotics due to thermodynamic forces. However, the underlying mechanisms warrant further investigation.

Filamentous phages may also promote antibiotic tolerance by slowing bacterial growth in ways that diminish the impact of antibiotics targeting cellular division. Pf production comes at a high metabolic cost to *P. aeruginosa*; Pf+ strains grow more slowly than Pf- strains in vitro [73,131,142,146]. Slow-growing “persister” phenotypes are a major contributor to antibiotic tolerance [147]. These effects may be clinically important. Burgener et al. found that Pf phages were associated with chronic *P. aeruginosa* infections and worse clinical outcomes in individuals with CF. Moreover, *P. aeruginosa* strains from patients with Pf phages detected in their sputum show increased antibiotic resistance and, over time, come to dominate the airways of individuals with CF [148]. Similarly, Pf+ strains of *P. aeruginosa* characterize chronic wound infections [149]. A recent modeling study examined how Pf comes to dominate in the CF lung and other environments. It suggested that antibiotic selection pressure is essential for promoting the dominance of Pf+ strains as in the absence of this, the high energetic cost of producing Pf phage would greatly favor Pf- over Pf+ strains of *P. aeruginosa* [150]. Together, these results suggest that Pf promotes antibiotic tolerance and may contribute to the selection of antibiotic-resistant mutants over time.

Filamentous phages are associated with drug resistance in other species. For instance, an agricultural pathogen, *Ralstonia solanacearum* uses an RSM1-like phage to acquire drug resistance at the cost of twitching motility [151,152,153]. Such mechanisms may be common among *Inoviridae* and their hosts since the production of these bacteriophages without bacterial lysis permits symbiotic relationships to evolve.

## 4. Lytic Phages and Antibiotic Susceptibility

Lytic or virulent phages infect bacteria and hijack host machinery for genomic replication and virion assembly. Lytic replication results in bacterial lysis, with progeny phages infecting new susceptible bacterial hosts [55].

**Figure 3:**
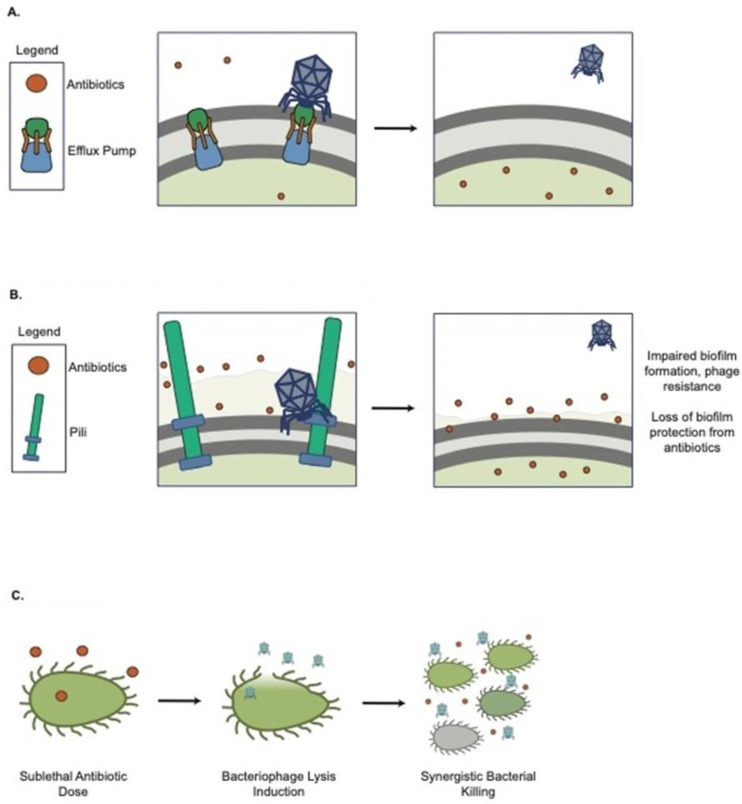
Lytic phages are used in combination with antimicrobial treatments against bacterial infection. (**A**) OMKO resistance increases antibiotic susceptibility; (**B**) DMS3vir resistance (loss of pili) impairs bacterial attachment; (**C**) Antibiotics trigger phage release and synergistic bacterial killing.

Bacteria have evolved a myriad of constitutive and inducible defense strategies against lytic phages [154], and these defenses can have direct implications for AMR. Constitutive defense against a phage is achieved by mutation or masking of phage receptors [154]. Surface modifications often come at a fixed but maladaptive and pleiotropic cost to the host bacteria [155], and they can have a direct effect on antibiotic resistance if the phage receptor is involved in AMR mechanisms. Chan et al. showed that *Pseudomonas* phage OMKO1 binds to the outer membrane porin M (OprM) component of the MexAB and MexXY multi-drug efflux systems of PAO1, which increases bacterial antibiotic susceptibility to drugs exported by this pathway [156] (Figure 3A). This strategy of choosing phages that bind to AMR-involved surface proteins is being exploited by some phage therapy companies. Phage infection poses a selective pressure for the bacteria to lose the AMR transporters and become sensitive to antibiotics. Surface modifications can also indirectly impact bacterial fitness and antibiotic tolerance [157,158]. Westra et al. demonstrated that *Pseudomonas aeruginosa* PA14 evolves immunity to phage DMS3vir under high-phage conditions through loss of the pilus [159], which can impair biofilm formation and, therefore, may reduce antibiotic tolerance [142,160,161,162] (Figure 3B). In addition, a recent paralleled evolution study suggested a trade-off in bacteria resistant to phage, which leads to a slow growth rate and reduced virulence [163].

There are indications that lytic phage and conventional antibiotics may act synergistically to kill bacteria (Figure 3C). The phrase phage antibiotic synergy (PAS) was first coined by Comeau et al. [164]. They found that certain antibiotics at sub-lethal concentrations stimulate virulent phage production in vitro, where sub-lethal cefotaxime can enhance uropathogenic *E. coli* isolate (MFP)’s phage production by 7-fold. Later, many studies, including Tagliaferri et al. [165], showed that various phages could be synergistic with different classes of antibiotics and enhance the eradication of bacteria. Particularly, phage and antibiotic combinations are efficacious in killing *P. aeruginosa* [166,167,168], *E. coli* [164,169,170], and *S. aureus* [171] in both planktonic and biofilm growth modes. Phage/antibiotic interactions can be synergistic, additive, or antagonistic. To facilitate the identification of these patterns, Liu et al. developed a new high-throughput method of screening phage and antibiotic interactions using real-time microtiter plate readouts. Using this approach, they reported that PAS combinations are both phage and antibiotic-specific [172].

PAS has also been efficacious in in vivo and clinical settings [173,174,175,176,177,178]. Various animal models have suggested that phage in combination with antibiotics can enhance the outcomes as well as reduce resistance development. Yilmaz et al. demonstrated a significant effect of PAS in a rat model of implant-associated *S. aureus* and *P. aeruginosa* infections. The combination therapy eradicated *S. aureus* biofilm [174]. Oeschlin et al. demonstrated that phage and ciprofloxacin were effective at rapidly eradicating bacteria and preventing the development of resistance in a mouse model of endocarditis [175]. Khawaldeh et al. described a single case of a 67 y/o woman with a recurrent, multi-drug resistant *P. aeruginosa* urinary tract infection. The patient was treated with a cocktail of six antipseudomonal phages, meropenem, and colistin. The infection resolved after 21 days, with no recurrence. It is unclear if there was any true synergy between the antibiotic and phage treatments, although phage therapy was successful at reducing bacterial burdens initially prior to the initiation of colistin [176]. Recently, after showing PAS in vitro [156], Chan et al. successfully employed PAS for the treatment of *P. aeruginosa* aortic graft infection with OMKO1 and continuous treatment of intravenous ceftazidime [177].

Moreover, temperate phages have drawn researchers’ interest due to their abundance in nature. Both natural and engineered temperate phages have been explored for therapy [179]. Recently, Al-Anany et al. demonstrated PAS by co-administration of temperate phage HK97 with sub-MIC ciprofloxacin resulting in an over 8-log bacterial burden reduction in vitro. However, concern has been raised by a recent PAS modeling study using either temperate or chronic phages, which suggests antibiotic resistance would likely develop [21].

Hence, antibiotics must be selected very carefully for PAS to avoid introducing unnecessary phage resistance. Recently, Dimitriu et al. used *P. aeruginosa* and DMS3vir as a model to demonstrate that bacteriostatic antibiotics, including chloramphenicol, tetracycline, erythromycin, and trimethoprim, can reduce bacterial growth and delay phage development to prompt bacterial acquisition of phage-derived novel spacers into host CRISPR array [178]. Their data suggest the importance of carefully selecting antibiotics in PAS to prevent the development of bacterial CRISPR immunity against lytic phage. Fortunately, studies have shown that bacteria resistant to phage tend to become less virulent and experience loss of fitness in host microenvironments [180,181]. In addition, Salazar et al. used a bioreactor to arise an evolved phage by directed evolution against the bacterial resistant isolates [180].

## 5. Conclusions

The data reviewed here suggest that lytic phage therapy may act synergistically with conventional antibiotics in ways that forestall the development or spread of AMR. In the same way that cocktails of antiretrovirals are used to prevent the emergence of resistance in HIV treatment, it is intriguing to imagine that cocktails of phages and conventional antibiotics, with careful selection, could have utility against AMR pathogens. This approach may have particular utility against biofilm infections or other hard-to-treat infections [182,183,184,185].

Most AMR is presumably due to transcriptional adaptation of bacteria against antibiotics. However, there are also indications that phage can contribute to both antibiotic resistance and tolerance. While phages are not the primary mechanism of AMR transfer, lysogenic phages can carry ARGs on prophage. Additionally, both lytic and lysogenic phages can transfer ARGs via general and lateral transductions. Filamentous phages can promote antimicrobial tolerance via antibiotics sequestration and perhaps slow down bacterial growth. Over time, this may promote the development of AMR by selecting for resistant clones/mutants. Currently, with the limited examples of clinical phage therapy, there is yet insufficient evidence to suggest that phage therapy can spread AMR. Nonetheless, care should be taken to avoid the possibility of spreading AMR when designing and testing phage therapy preparations for clinical use.

Many questions remain. It is important to understand the relative contributions of phages versus other MGEs to AMR spread and the evolutionary pressures that mediate these. Similarly, it would be interesting to understand how lytic and lysogenic phages differ with regard to ARG transfer, given their distinct impacts on their bacterial hosts. More work is needed to clarify how such costs influence the development of antibiotic tolerance and resistance.

The intersection between antibiotics and bacteriophages is a frontier in AMR research that is ripe for exploration. The potential dividends of such research are great, with potential benefits for many patients.

## Data Availability

Not applicable.

## Abbreviations

AMR	Antimicrobial resistance
MIC	Minimum inhibitory concentration
XDR-TB	Extensively drug-resistant tuberculosis
VRSA	Vancomycin resistance genes-resistant *Staphylococcus aureus*
ARG	Antibiotic resistance genes
MGE	Mobile genetic elements
ICEs	Integrative and conjugative elements
CF	Cystic fibrosis
PAS	Phage antibiotic synergy
